# Incidence of Fetal Macrosomia in the Cantonal Hospital Zenica: Retrospective Analysis (2020-2024)

**DOI:** 10.7759/cureus.96851

**Published:** 2025-11-14

**Authors:** Faris Kazic, Enid Nakicevic, Rasim Iriskic

**Affiliations:** 1 Obstetrics and Gynecology, Cantonal Hospital Zenica, Zenica, BIH

**Keywords:** apgar score, birth weight, delivery mode, fetal macrosomia, parity

## Abstract

Aim

The aim was to determine the incidence and temporal trends of fetal macrosomia at Cantonal Hospital Zenica between 2020 and 2024.

Methods

A retrospective analysis of all deliveries (n = 11,117) from 2020-2024 was performed. Macrosomia was defined as birth weight ≥4,000 g. Descriptive statistics, trend analysis, and simple linear regression relating total births and macrosomic births were used. Neonatal outcomes included mean birth weight and one‑ and five‑minute Apgar scores; parity, maternal age groups, and mode of delivery were examined. Chi‑square tests assessed yearly distribution changes.

Results

Of 11,117 deliveries, 1,625 (14.6%) were macrosomic. Annual macrosomia counts declined from 369 (2020) to 295 (2024) while total deliveries fell from 2,478 (2020) to 2,083 (2023) and rose to 2,118 (2024). Mean macrosomic birth weight was 4,263.0 g (annual range 4,254.1-4,270.8 g). Mean one‑minute and five‑minute Apgar scores were 7.89 and 8.80, respectively; 5.91% of neonates had one‑minute Apgar <7 and 1.48% had five‑minute Apgar <7. Primiparas accounted for 38.22% of macrosomic births (621/1,625) and experienced a high cesarean rate (62.32%). Simple linear regression showed a strong association between total and macrosomic births (R = 0.973; R2 = 0.947; p = 0.0052), though interpretation of the model slope requires caution. Yearly changes in parity distribution, maternal age groups, and mode of delivery were not statistically significant (chi‑square tests, all p>0.05).

Conclusions

Macrosomia constituted a substantial and relatively stable proportion (~14.6%) of births at this referral center, with generally favorable immediate neonatal outcomes and a high cesarean proportion among primiparas. Findings support continued emphasis on antenatal metabolic risk optimization, individualized delivery planning, and maintenance of intrapartum and neonatal emergency preparedness. Study limitations include the short (five‑year) time series and lack of individual covariates (BMI, GDM status, gestational age), which constrain causal inference and adjusted analyses.

## Introduction

Fetal macrosomia denotes excessive fetal growth and is most defined as birth weight ≥4,000 g, irrespective of gestational age [1]. Some authors and clinical guidelines use alternative thresholds (for example ≥4,500 g) to differentiate moderate from severe macrosomia [2]. The definition remains debated because birthweight distributions vary by ethnicity, geography, and population characteristics; nevertheless, macrosomia is widely recognized as an important perinatal concern because of its association with adverse short‑ and long‑term maternal and neonatal outcomes [3,4].

The prevalence of fetal macrosomia varies worldwide, typically ranging from approximately 5% to 20% of live births depending on the studied population and the threshold applied [4,5]. In high‑income settings, where maternal obesity and gestational diabetes mellitus (GDM) are increasingly common, macrosomia rates are often higher [6]. Macrosomia is a multifactorial condition with maternal and fetal determinants. The most consistently reported maternal risk factors include pre‑pregnancy obesity, GDM, multiparity and advanced maternal age [6,7]. Fetal and placental factors such as male sex, post‑term gestation (>40 weeks) and increased placental size also contribute to risk [8].

Macrosomic infants face higher risks of shoulder dystocia, birth trauma, neonatal hypoglycemia, and neonatal intensive care admission, while affected mothers have increased rates of cesarean delivery, severe perineal injury, and postpartum hemorrhage [9-11]. Accurate, locally relevant estimates of macrosomia incidence are therefore critical to inform antenatal screening, delivery planning and healthcare resource allocation. Despite numerous regional and international studies, local epidemiologic data from Bosnia and Herzegovina are limited. Cantonal Hospital Zenica is a major obstetric referral center for the Zenica‑Doboj Canton, yet the incidence and outcomes of fetal macrosomia in this population have not been systematically reported.

The primary objective was to describe the incidence and immediate neonatal outcomes of fetal macrosomia (birthweight ≥4,000 g) among term deliveries at Cantonal Hospital Zenica, Zenica‑Doboj Canton, Bosnia and Herzegovina, during 2020-2024; secondary objectives were to compare mode of delivery by parity, assess temporal trends, and report key neonatal outcomes.

## Materials and methods

This study was designed as a retrospective, descriptive-analytical review of delivery records from the Department of Gynecology and Obstetrics, Cantonal Hospital Zenica, Zenica, Bosnia and Herzegovina. Cantonal Hospital Zenica Ethics Committee issued approval 00-03-35-421-12/25. The study covered a five-year period from January 1, 2020 to December 31, 2024. The hospital is a secondary referral center providing obstetric care for the Zenica-Doboj Canton.

Data were extracted from the hospital’s official electronic delivery registry and verified against paper delivery logs by two researchers. The study population consisted of all women who delivered neonates with a birthweight of ≥4000g during the study period (2020-2024). Only term pregnancies, defined as deliveries between 37+0 and 41+6 weeks of gestation, were included. Exclusion criteria were preterm births (<37 weeks), multiple gestations, fetal congenital anomalies, non‑viable births, and duplicate or incomplete records. For each patient, the following variables were extracted: maternal age, parity, mode of delivery (spontaneous vaginal or cesarean section), and neonatal outcomes including Apgar scores at one and five minutes.

Data analysis was performed using descriptive and inferential statistical methods. Descriptive statistics included calculation of means, standard deviations, minimum and maximum values for continuous variables, and absolute and relative frequencies for categorial variables. The incidence of macrosomic deliveries was expressed as annual and overall percentages. Temporal trends in the number of total deliveries and macrosomic deliveries between 2020-2024 were examined using simple linear regression analysis, with calculation of regression coefficients and coefficients of determination (R2). The strength and direction of association between the number of total deliveries and the number of macrosomic deliveries were additionally assessed using Pearson’s correlation coefficient (r).

Differences in the distribution of primiparous and multiparous women across study years were evaluated using the chi-square (ꭓ2) test for categorical data. Mean birthweights of macrosomic neonates were compared between years; given the presence of four independent groups, one-way analysis of variance (ANOVA) was applied to test for statistically significant differences.

All statistical tests were two-tailed, and a p-value <0.05 was considered statistically significant.

## Results

**Figure 1:**
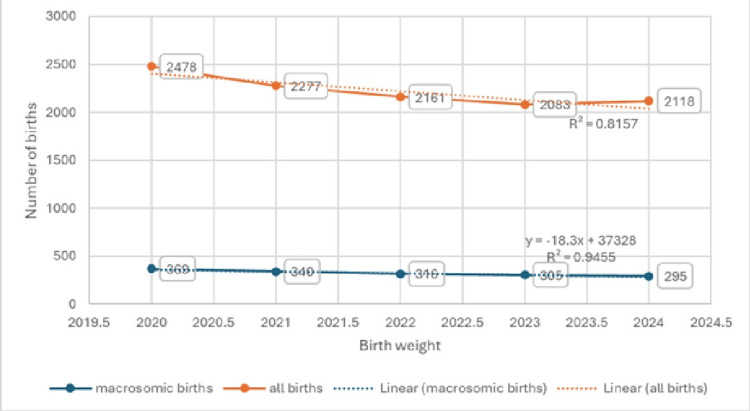
Total number of deliveries and the number of macrosomic deliveries per year with linear trend lines.

During the observation period from 2020 to 2024, there was a total of 11117 deliveries. On average, 14.6% (1625 out 11117) were macrosomic deliveries (Table 1). The proportion of macrosomic deliveries in relation to the total number of deliveries during the observed period was approximately equal across years. These data are presented in Figure 1.

The graph shows a slight downward trend in macrosomic births, with an R^2 of 0.9455, while the trend for total births declines somewhat more steeply, with an R^2 of 0.98157. The plotted data indicate a steady year‑to‑year decrease in the number of macrosomic births. Total births also decline, exhibiting a large standard deviation (SD = 160.01), whereas the variability in macrosomic births is small (SD = 29.75).

The maximum number of total births was 2478 and the minimum was 2083. The highest count of macrosomic births was 369 and the lowest was 295. These data are presented in Table 1.

**Table 1:** Descriptive statistics for macrosomic and total births, 2020–2024

Variable	Mean	Maximum	Minimum	Standard deviation
Macrosomic births	325	369	295	29.75
Total births	2223.4	2478	2083	160.01

Regression analysis revealed a statistically significant association between total births and macrosomic births: for each 1% increase in total births, the number of macrosomic births decreased on average by 18.3%. The coefficient of determination was 0.947, indicating a very strong relationship by Chadokov’s scale; thus, the simple linear regression model is highly representative (R = 0.973; R2 = 0.947; p = 0.0052). These data are presented in Table 2. 

**Table 2:** Regression analysis of macrosomic and total births for the period 2020–2024

Measurement	Value
Pearson correlation coefficient	0.973
Coefficient of determination	0.947
Standard error	7.8797
Sample size (number of observations)	5

The average proportion of primiparas among macrosomic births was 38.22% (621 of 1625). The percentage of primiparas decreased each year except in 2024, when it reached 40.0% (118 of 295), the maximum for the observed period; the lowest proportion was in 2022 at 36.71% (116 of 316). These data are presented in Table 3.

**Table 3:** Distribution of primiparas and multiparas among macrosomic births, 2020–2024 N - number of births

Year	Primiparas		Multiparas		N
2020	144	39.02%	225	60.98%	369
2021	128	37.65%	212	62.35%	340
2022	116	36.71%	200	63.29%	316
2023	115	37.70%	190	62.30%	305
2024	118	40.00%	177	60.00%	295
S	621	38.22%	1004	61.78%	1625

**Figure 2:**
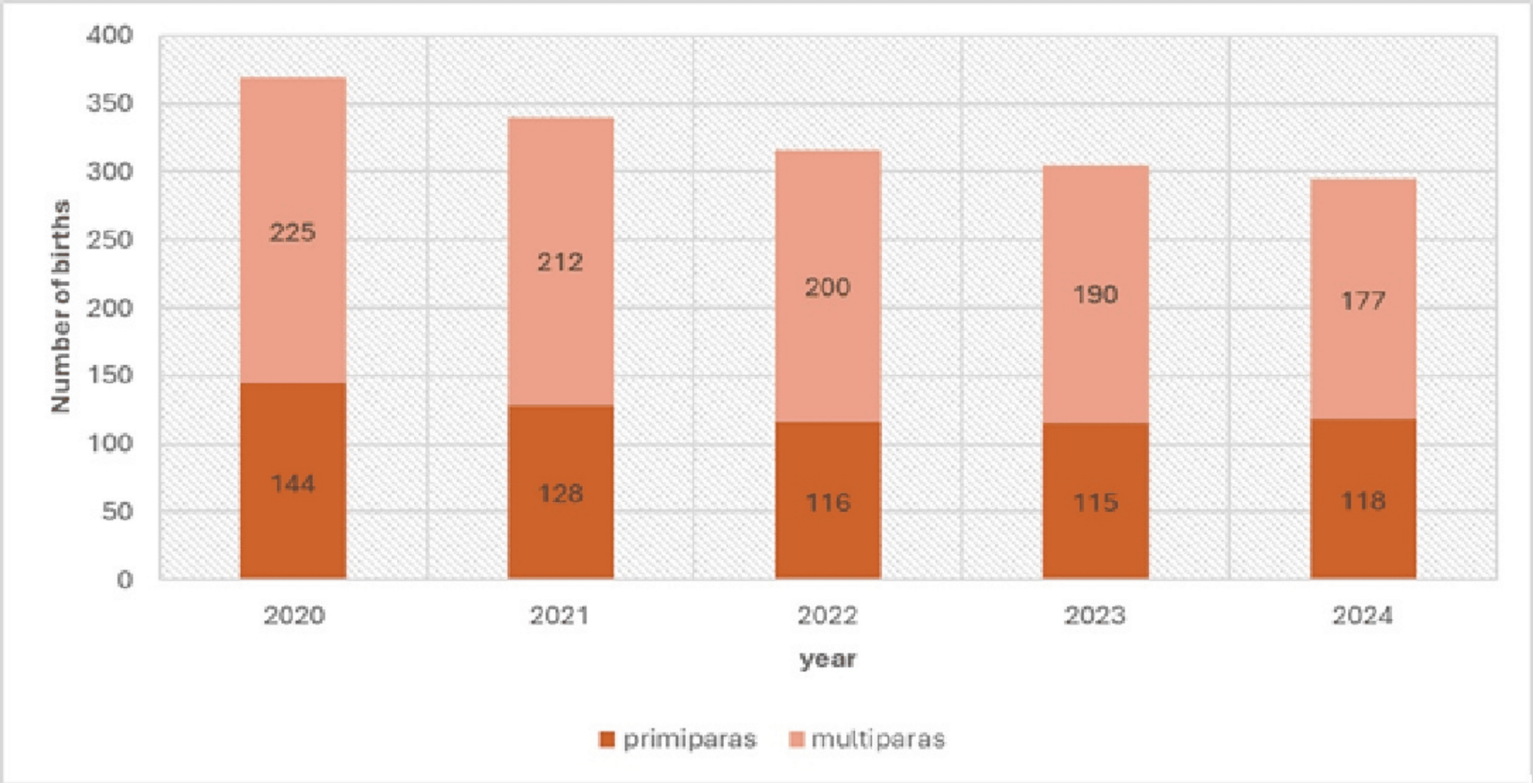
Distribution of primiparas and multiparas among macrosomic births, 2020–2024

Observed annual differences in the proportion of primiparas versus multiparas varied but were small and not statistically significant (χ2 = 0.8841; p = 0.9268; df = 4; χ2 critical = 9.4877), which is presented in Figure 2.

**Figure 3:**
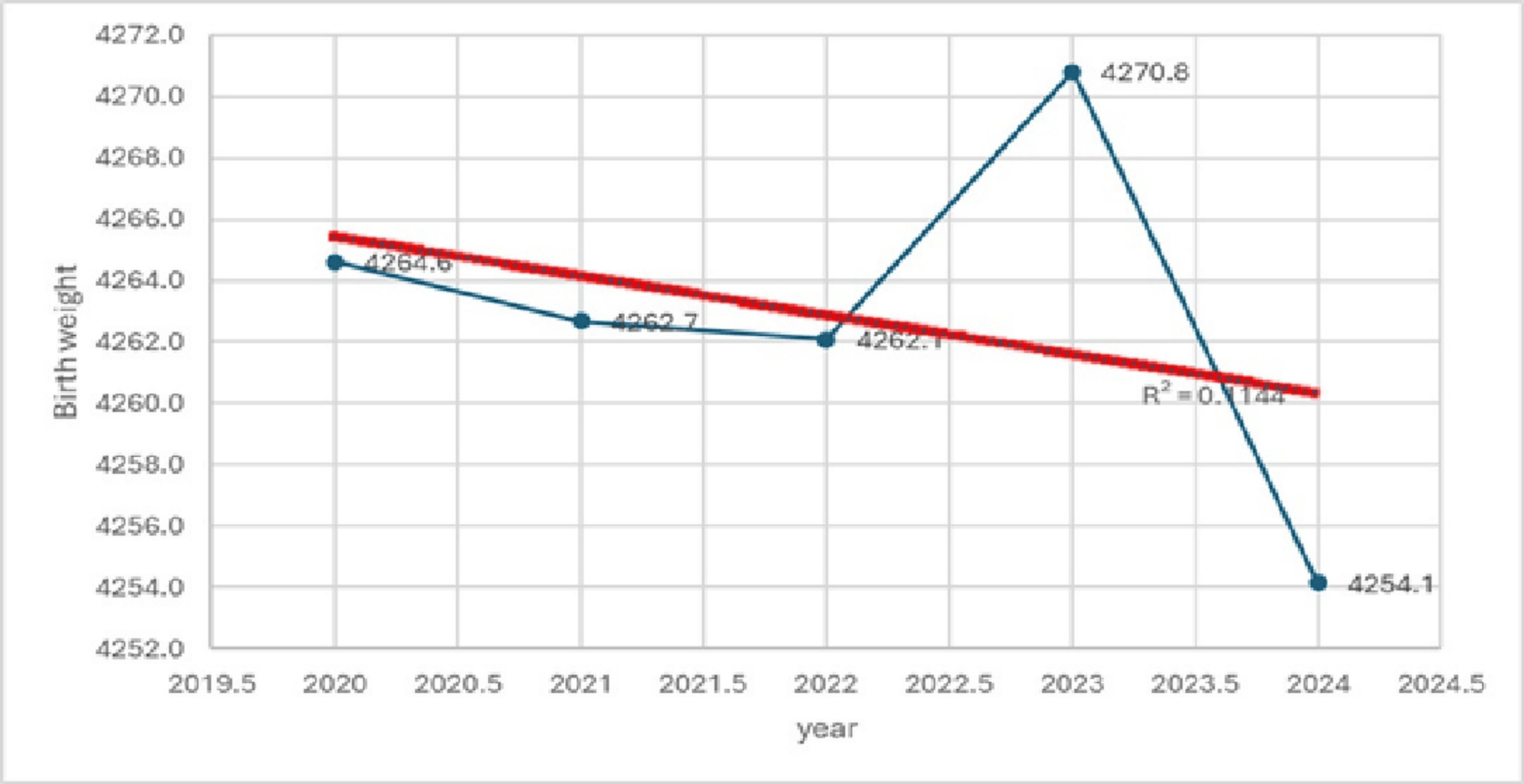
Trend in mean birth weight, 2020–2024

The mean birth weight of neonates with macrosomia over the study period was 4263.0 g. The lowest mean birth weight occurred in 2024 (4254 g), and the highest was in 2023 (4270.8 g). These data are presented in Table 4 and Figure 3.

**Table 4:** Mean birth weight of neonates among macrosomic births, 2020–2024 N - number of births

Year	Mean birth weight	N
2020	4264.6	369
2021	4262.7	340
2022	4262.1	316
2023	4270.8	305
2024	4254.1	295
S	4263.0	1625

Neonatal health status was categorized by grouped Apgar score ranges: scores ≤4 indicate the neonate requires monitoring and assistance; scores 5-7 indicate poorer condition and the need for supportive care; and scores 8-10 indicate the neonate is in good health.

The mean one‑minute Apgar score for the study period was 7.89 (lowest mean: 7.80 in 2021; highest mean: 8.10 in 2023). The mean percentage of neonates with a one-minute Apgar score less than 7 over the observation period was 4.76%, with the lowest average of 4.54% in 2021 and the highest of 5.00% in 2023. The percentage of neonates with a one-minute Apgar score less than 7 during the study period was 5.91%, with the lowest percentage of 1.64% in 2023 and the highest of 8.14% in 2024. These data are presented in Table 5.

**Table 5:** Mean one-minute Apgar score and percentage of neonates with one minute Apgar <7, 2020-2024 N - number of births

Year	One-minute Apgar <7			One-minute Apgar	
	mean	N		mean	N
2020	4.89	18	4.88%	7.90	369
2021	4.54	24	7.06%	7.80	340
2022	4.64	25	7.91%	7.79	316
2023	5.00	5	1.64%	8.10	305
2024	4.96	24	8.14%	7.87	295
S	4.76	96	5.91%	7.89	1625

The mean five‑minute Apgar score for the study period was 8.80, with the lowest average of 8.74 in 2022 and the highest of 8.92 in 2023. The mean percentage of neonates with a five-minute Apgar score less than 7 over the study period was 4.87%, with the highest average of 5.88% in 2022. In 2023, there were no cases of neonates with a five-minute Apgar score below 7, which is reflected as the lowest average. The percentage of neonates with a five-minute Apgar score less than 7 during the study period was 1.48%, with the lowest percentage of 0.0% in 2023 and the highest of 2.53% in 2022. These data are presented in Table 6.

**Table 6:** Mean five-minute Apgar score and percentage of neonates with five-minute Apgar <7, 2020–2024 N - number of births

Year	Five-minute Apgar <7			Five-minute Apgar	
	mean	N		mean	N
2020	5.50	4	1.08%	8.81	369
2021	2.67	6	1.76%	8.75	340
2022	5.88	8	2.53%	8.74	316
2023	0.00	0	0.00%	8.92	305
2024	5.33	6	2.03%	8.79	295
S	4.87	24	1.48%	8.80	1625

A one-minute Apgar score ≤4 was observed in 2.3% of neonates (30 of 1625). By year, the smallest proportion of neonates with a one‑minute Apgar score ≤4 was in 2023 (0.66%), and the largest was in 2024 (2.37%), with an overall mean of 2.30%. The smallest proportion of neonates with one‑minute Apgar scores of 5-7 was in 2023 (14.75%), and the largest was in 2024 (21.36%), with an overall mean of 18.03%. The smallest proportion of neonates with one‑minute Apgar scores of 8-10 was in 2024 (76.27%), and the largest was in 2023 (84.59%), with a yearly mean of 80.12%. These data are presented in Table 7.

**Table 7:** Neonatal health indicators by Apgar score, 2020–2024 N - number of births

Year	Apgar score						N
	4 or lower		5 - 7		8 or higher		
2020	7	1.90%	60	16.26%	302	81.84%	369
2021	7	2.06%	62	18.24%	271	79.71%	340
2022	7	2.22%	63	19.94%	246	77.85%	316
2023	2	0.66%	45	14.75%	258	84.59%	305
2024	7	2.37%	63	21.36%	225	76.27%	295
S	30	2.30%	293	18.03%	1302	80.12%	1625

When primiparas given birth to a macrosomic neonate are classified by age, there are few women over 40 years old (a total of seven). A slight increasing trend is observed in the number of primiparas over 40 years. The most numerous among primiparas under 20 years old were in 2023, with nine cases, while the fewest were in 2022, with three cases.

The trend line for primiparas aged 20-30 years shows a slight decline, with a determination coefficient of 0.3341, as does the trend line for primiparas aged 30-40 years, with a coefficient of 0.4375. The differences in these trends indicate that the number of younger primiparas (20-30 years) is decreasing at a faster rate than those aged 30-40 years.

**Figure 4:**
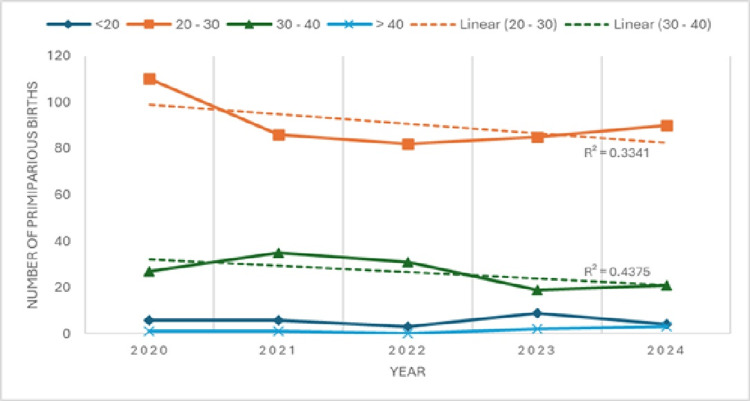
Distribution of primiparas by age group, 2020–2024

The average age of primiparas increased over the observation years. The differences between primiparas younger than 30 years and those older than 30 years are small and not statistically significant (χ2 = 5.772984; p = 0.216756; df = 4; χ2 critical = 9.4877). These data are presented in Table 8 and Figure 4.

**Table 8:** Distribution of primiparous mothers by age group, 2020–2024 N - number of births

Year	<20	20-30	30-40	>40	mean	N
2020	6	110	27	1	25.72	144
2021	6	86	35	1	26.53	128
2022	3	82	31	0	26.86	116
2023	9	85	19	2	26.05	115
2024	4	90	21	3	26.59	118
S	28	453	133	7	26.33	621

When multiparas who gave birth to a macrosomic neonate were analyzed by age group, the number of younger multiparas (<20 years) was small (n = 6), while multiparas >40 years showed a slight increasing trend. Multiparas >40 years were most frequent in 2022 (n = 11) and least frequent in 2021 (n = 3).

The trend line for multiparas aged 20-30 years shows a steady decline (R2 = 0.8302), as does the trend for multiparas aged 30-40 years (R2 = 0.9944). These trends indicate a faster decrease in the number of multiparas aged 30-40 years compared with those aged 20-30 years.

**Figure 5:**
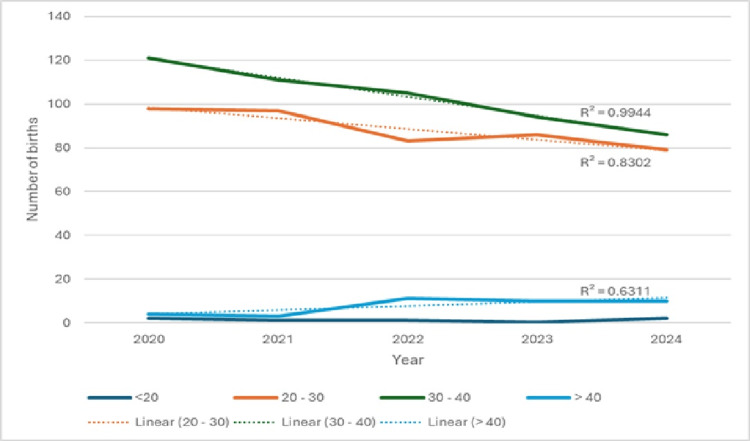
Distribution of multiparas by age group, 2020–2024

Mean maternal age of multiparas increased over the observation period. The observed differences between multiparas <30 years and ≥30 years were small and not statistically significant (χ2 = 0.900578; p = 0.924478; df = 4; χ2 critical = 9.4877). These data are presented in Table 9 and Figure 5.

**Table 9:** Distribution of multiparous mothers by age group, 2020–2024 N - number of births

Year	<20	20-30	30-40	>40	mean	N
2020	2	98	121	4	30.05	225
2021	1	97	111	3	30.09	212
2022	1	83	105	11	30.69	200
2023	0	86	94	10	30.74	190
2024	2	79	86	10	30.77	177
S	6	443	517	38	30.44	1004

The percentage of vaginal deliveries among primiparas was highest in 2020 (47.22%) and lowest in 2022 (32.76%), with a mean of 37.68%. The percentage of cesarean deliveries among primiparas was highest in 2022 (67.24%) and lowest in 2020 (52.78%), with an overall mean of 63.32%. These data are presented in Table 10. 

**Table 10:** Distribution of vaginal and cesarean deliveries among primiparas, 2020–2024 N - number of births

Year	Vaginal birth		Cesarean section		N
2020	68	47.22%	76	52.78%	144
2021	43	33.59%	85	66.41%	128
2022	38	32.76%	78	67.24%	116
2023	43	37.39%	72	62.61%	115
2024	42	35.59%	76	64.41%	118
S	234	37,68%	387	62.32%	621

**Figure 6:**
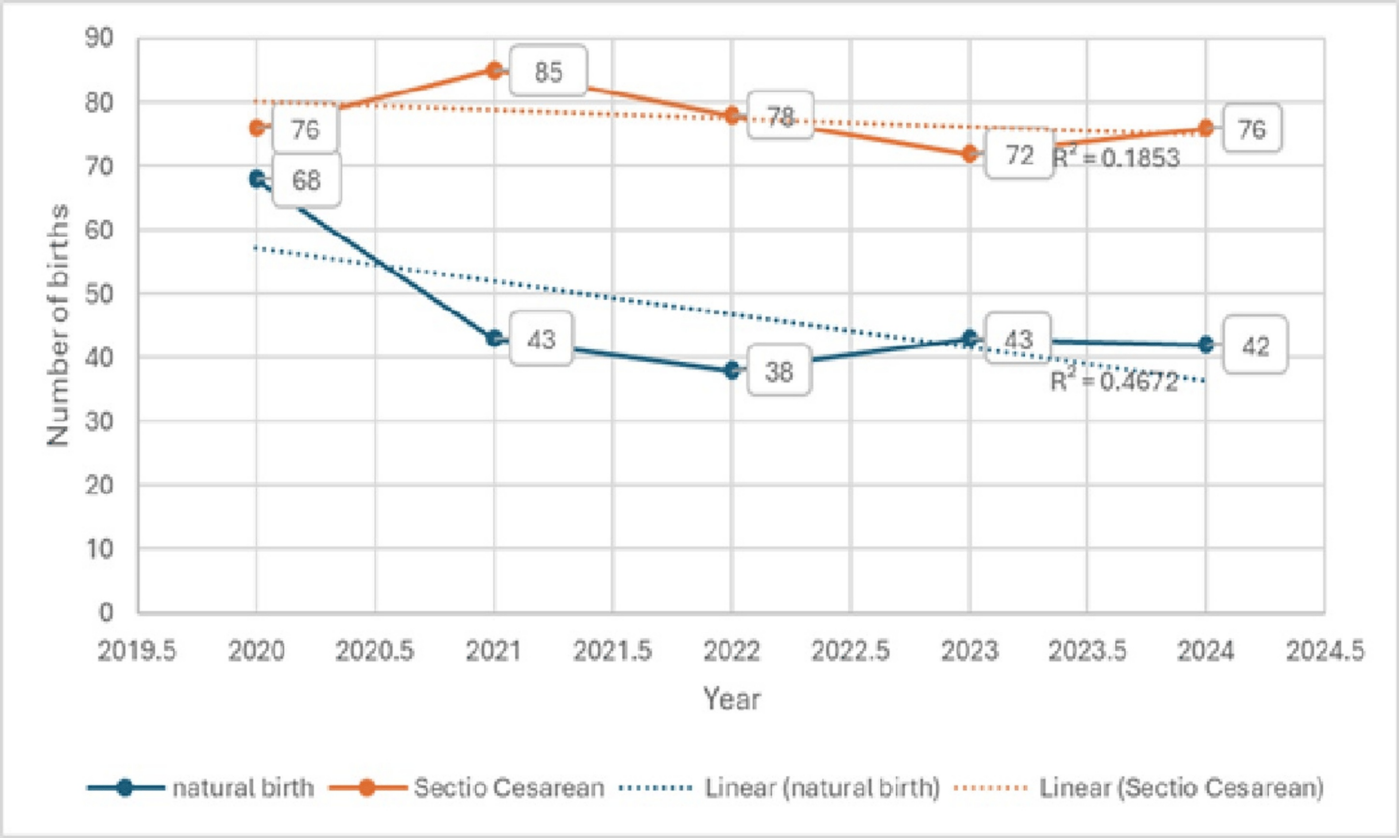
Distribution of vaginal and cesarean deliveries among primiparas, 2020–2024

Over the study period the proportion of vaginal deliveries among primiparas decreased year by year, while the proportion of cesarean deliveries increased; however, this trend was not statistically significant (χ2 = 7.913139; p = 0.094812; df = 4; χ2 critical = 9.4877). These data are presented in Figure 6.

The trend line for vaginal deliveries shows a decline (R² = 0.4672); while percentage‑wise there was an approximate 10% increase in cesarean deliveries among primiparas over the study period, this change did not reach statistical significance (χ2 = 7.913, p = 0.095).

## Discussion

This study analyzed macrosomic births among 11117 deliveries from 2020 through 2024, providing descriptive, trend, and inferential data on macrosomia incidence, neonatal outcomes (Apgar), parity, maternal age groups, and mode of delivery. Overall, 1625 (14.6%) births met the macrosomia definition used in this cohort. The dataset permits several observations with clinical and public‑health relevance: a modest absolute decline in both total and macrosomic births over time with very high model fit for the association between the two variables, a relatively stable proportion of macrosomia among all births, generally favorable neonatal outcomes by Apgar with few severe early depressions, parity and maternal‑age distributions that changed minimally and without statistically significant shifts, and a predominance of cesarean delivery among primiparas with a non‑significant upward trend. Across the five years, total deliveries decreased from 2478 (2020) to 2083 (2023) with a modest rebound to 2118 in 2024, while macrosomic births decreased from 369 (2020) to 295 (2024). The simple linear regression demonstrated a strong positive Pearson correlation (R = 0.973) and coefficient of determination R2 = 0.947 (p = 0.0052), indicating that variation in total births explains approximately 94.7% of the variance in observed macrosomic births in this sample; however, the regression statement that “for each 1% increase in total births macrosomic births decrease by 18.3%” appears counterintuitive (likely reflecting model scaling/interpretation or a negative slope expressed in percent terms), so causal claims should be avoided. These changes likely reflect short‑term shifts in referral patterns and service utilization (notably during the COVID‑19 pandemic years), small year‑to‑year demographic variation, and possible secular changes in antenatal care or risk factor prevalence (eg, glycemic control, maternal BMI). Because our data lack individual‑level timing, migration, and antenatal covariates, we cannot attribute causation; the parallel decline in total and macrosomic births suggests that overall birth volume influenced macrosomia counts more than a true change in macrosomia prevalence.

Comparisons with population data and prior studies suggest mixed dynamics. Many high‑income settings have reported either rising macrosomia rates associated with increasing maternal obesity and diabetes, or stable rates where obstetric management, glycemic control, and population characteristics offset those risks [3-5]. The current cohort’s stable percentage of macrosomia (~14-15%) across years indicates no abrupt epidemiologic shift and may reflect balanced changes in maternal risk factors, referral patterns, or clinical practices (e.g., antenatal screening, glycemic control, induction or planned cesarean policies for large fetuses) [6,10,12-16]. The observed smaller standard deviation for macrosomic births (SD = 29.75) relative to total births (SD = 160.01) further supports that macrosomia counts varied less year‑to‑year despite fluctuations in overall deliveries.

The mean birth weight among macrosomic neonates was 4263.0 g (range of annual means 4254.1-4270.8 g). The annual variation was minimal (<20 g between minimum and maximum annual means), consistent with a relatively homogeneous macrosomic population with limited temporal weight shifts. In literature, mean weights in macrosomic cohorts commonly range from approximately 4000 to 4400 g depending on the population, nutrition, ethnicity, and rates of maternal hyperglycemia; the present values fall within expected bounds [7,13]. Clinically, stable mean birth weights suggest consistent antenatal detection and management thresholds and no sudden change in the underlying determinants (e.g., maternal glycemia, obesity trends) during the observation window.

Apgar results were generally reassuring, with mean one‑minute Apgar = 7.89 and mean five‑minute Apgar = 8.80. The overall proportion of neonates with one‑minute Apgar <7 was 5.91%, and with five‑minute Apgar <7 was 1.48%. Only 2.3% of macrosomic neonates had one‑minute Apgar ≤4 (30/1625). Yearly variation existed: the lowest one‑minute Apgar <7 percentage occurred in 2023 (1.64%) and the highest in 2024 (8.14%); for five‑minute Apgar <7 the highest observed annual percentage was 2.53% in 2022 while 2023 had 0%. These findings indicate that despite macrosomia-associated risks (shoulder dystocia, birth asphyxia, trauma), most neonates in this cohort achieved satisfactory immediate postnatal adaptation.

Existing studies show that macrosomia increases the risk of shoulder dystocia and birth trauma, which in turn can negatively affect immediate Apgar scores and neonatal outcomes [8,11]. However, many contemporary obstetric services mitigate these risks via intrapartum management (e.g., operative deliveries when indicated, skilled shoulder dystocia maneuvers, neonatal resuscitation availability) leading to low overall proportions of severe Apgar depression as seen here [11,17]. The temporal decrease in poor one‑minute Apgar scores until 2023, with a rise in 2024, invites scrutiny of year‑specific practices, staffing, case mix (e.g., proportion of diabetic pregnancies), or data coding; nonetheless, five‑minute Apgar scores remained consistently high across study years, implying effective immediate neonatal stabilization when needed.

Primiparas represented 38.22% of macrosomic births (621/1625) and multiparas 61.78%. The share of primiparas fluctuated modestly but did not change significantly over time (χ2 = 0.8841; p = 0.9268). Age stratification among primiparas showed a small absolute number of mothers >40 years (n = 7), a slight increase in that group over time, and a modest decline in the 20-30 age group (trend R2 = 0.3341) relative to 30-40 (R2 = 0.4375). For multiparas a decline was most pronounced in the 30-40 group (R2 = 0.9944) while the 20-30 group also declined (R2 = 0.8302). These results indicate only gradual demographic shifts without statistically significant changes in the proportion of younger versus older primiparas/multiparas (primipara χ2 = 5.773; p = 0.2168; multipara χ2 = 0.9006; p = 0.9245).

In comparison, many populations have shown increasing maternal age at first birth over recent decades, which can influence macrosomia risk through increased prevalence of comorbidities (obesity, diabetes) and gestational complications [18]. The small magnitude of age changes here suggests limited impact on macrosomia trends during the five‑year window; however, even modest age shifts could have clinical implications if coupled with rising metabolic risk prevalent in many populations.

Primiparas delivered vaginally in 37.68% of macrosomic births and by cesarean section in 62.32% (234 vaginal, 387 cesareans of 621). The proportion of vaginal deliveries was highest in 2020 (47.22%) and lowest in 2022 (32.76%), while cesarean peaked in 2022 (67.24%). The trend in vaginal deliveries showed a decline (R2 = 0.4672); the cesarean delivery trend had a smaller R2 (0.1853) though overall cesarean rates among primiparas were high. The interannual increase in cesarean deliveries may reflect clinician caution when managing primiparas with suspected macrosomia because of the elevated risk of intrapartum complications, fear of shoulder dystocia, medico‑legal considerations, patient preference, or institutional policies [9,19,20]. International guidance advises individualized decision‑making balancing fetal size estimates, maternal pelvic adequacy, and comorbidities; many centers report rising primary cesarean rates for suspected macrosomia despite limited evidence that elective cesarean for estimated large fetuses improves long‑term outcomes except in extreme cases [15,16].

The observed macrosomia prevalence (~14.6%) aligns with reported ranges in many settings where prevalence varies broadly (approx. 5-20% depending on definitions and population risks) [4,5]. Studies linking increasing maternal obesity and diabetes to rising macrosomia highlight the importance of metabolic risk management; conversely, some centers have maintained stable macrosomia rates because of improved antenatal care and obstetric management [3,12]. The high cesarean proportion among primiparas is consistent with global trends toward increased primary cesarean deliveries for suspected large fetuses, driven by risk aversion and differing institutional thresholds [9,20]. The low five‑minute Apgar rates and modest proportion of severe early depressions compare favorably with reports where high-quality intrapartum care reduces immediate adverse neonatal outcomes despite the mechanical risks of macrosomia [11].

The study’s findings carry several clinical implications. Continued emphasis on antenatal identification and optimization of maternal metabolic risk is warranted because macrosomia prevalence remained substantial (~14.6%); targeted interventions for gestational diabetes screening, tighter glycemic control, nutritional counseling, and weight management before and during pregnancy could reduce macrosomia‑related morbidity [6-10]. Delivery planning should remain individualized: the high cesarean proportion among primiparas suggests a low threshold for operative delivery when macrosomia is suspected yet given the known maternal risks of cesarean and uncertain benefit for moderate‑sized macrosomic fetuses, clinicians should discuss risks and benefits with patients and consider local outcomes (for example, the low five‑minute Apgar rates here) when forming policies [20]. Maintaining robust intrapartum and neonatal resuscitation readiness is important, as the low rates of severe five‑minute Apgar depression indicate effective immediate care; ongoing training in shoulder dystocia maneuvers, simulation, and neonatal resuscitation should be preserved [8,11]. Finally, temporal fluctuations (notably the higher proportion of one‑minute Apgar <7 in 2024) warrant local audit to determine whether changes in clinical practice, case mix, staffing, or data collection contributed to year‑specific differences.

The study has several important limitations that must be acknowledged. Its observational, ecological time‑series design with only five annual points limits causal inference and the stability of trend estimates, and high R and R2 values in such short series may reflect collinearity and limited degrees of freedom rather than robust, generalizable associations. The dataset lacks key individual covariates - pre‑pregnancy BMI, gestational diabetes status, hypertensive disorders, maternal smoking, ethnicity, estimated fetal weight, induction rates, and details of obstetric management - so confounding could not be addressed. The reported regression interpretation that “for each percent increase in total births, macrosomic births decrease by 18.3%” suggests possible model scaling or direction ambiguity; the regression results and model specification (counts versus percentages or log transformations) should therefore be re‑examined. Findings derive from a single institution or region and may not generalize to populations with different maternal characteristics, healthcare systems, or clinical practices. Finally, small yearly counts in some subgroups (for example mothers >40 years and neonates with Apgar ≤4) reduce precision and increase susceptibility to chance fluctuations.

## Conclusions

The present analysis provides a comprehensive local description of the burden of fetal macrosomia and associated immediate outcomes at a major referral center in Bosnia and Herzegovina over a recent five‑year interval. Macrosomia remained a stable and substantial component of the obstetric workload, consuming consistent clinical resources for antenatal assessment, intrapartum management and neonatal care. Neonatal adaptation outcomes were largely reassuring: the majority of macrosomic infants achieved satisfactory one‑ and five‑minute Apgar scores, severe early depression was uncommon, and rates of five‑minute Apgar <7 remained low across the study period, suggesting effective intrapartum management and prompt neonatal stabilization when needed. Nevertheless, primiparas with macrosomia experienced a disproportionately high rate of operative delivery, reflecting clinical risk‑avoidant decision making in the context of suspected large fetuses and underscoring the trade‑offs between minimizing neonatal complications and exposing mothers to the immediate and longer‑term risks of cesarean delivery.

These findings reinforce the need for sustained emphasis on antenatal metabolic risk optimization through systematic identification and management of gestational diabetes, preconception and antenatal weight‑management programs, and targeted nutritional counseling. Such preventive strategies can reduce the incidence of excessive fetal growth and thereby lower both neonatal morbidity and the pressure toward operative delivery. At the same time, delivery planning should be evidence‑informed and individualized, incorporating accurate estimation of fetal size, maternal pelvic assessment, prior obstetric history and patient preferences; multidisciplinary counseling and shared decision‑making may help balance neonatal safety with avoidance of unnecessary maternal surgical morbidity.

Maintaining obstetric and neonatal emergency readiness is essential: continued training in shoulder dystocia maneuvers, regular simulation of obstetric emergencies, standardized intrapartum protocols for suspected macrosomia and robust neonatal resuscitation capacity likely contributed to the favorable Apgar profile observed and must be preserved. Equally important is improving routine data capture to include key maternal and fetal covariates (pre‑pregnancy BMI, GDM status and control, gestational age, estimated fetal weight, induction and labor management, fetal sex and neonatal complications). Enhanced data systems would permit risk‑adjusted analyses, enable monitoring of intervention effectiveness, and support development of locally tailored clinical guidelines.

Finally, local audit and quality‑improvement initiatives should examine year‑specific fluctuations in neonatal adaptation metrics and operative delivery practices to identify modifiable contributors such as changes in case mix, staffing, clinical protocols or diagnostic thresholds. Structured audits, feedback loops and targeted training can ensure that care pathways optimize neonatal outcomes while minimizing unnecessary cesarean deliveries and their downstream maternal risks.
